# Efficacy of Targeted Temperature Management after Pediatric Cardiac Arrest: A Meta-Analysis of 2002 Patients

**DOI:** 10.3390/jcm10071389

**Published:** 2021-03-30

**Authors:** Wojciech Wieczorek, Jarosław Meyer-Szary, Milosz J. Jaguszewski, Krzysztof J. Filipiak, Maciej Cyran, Jacek Smereka, Aleksandra Gasecka, Kurt Ruetzler, Lukasz Szarpak

**Affiliations:** 1 Department of Emergency Medicine, Medical University of Warsaw, 02-091 Warsaw, Poland; w.wieczorek@easyrescue.pl; 2 Polish Society of Disaster Medicine, 50-345 Warsaw, Poland; jacek.smereka@umed.wroc.pl; 3 Department of Paediatric Cardiology and Congenital Heart Diseases, Medical University of Gdansk, 80-211 Gdansk, Poland; gray@gumed.edu.pl; 4 1st Department of Cardiology, Medical University of Gdansk, 80-211 Gdansk, Poland; mjaguszewski@gumed.edu.pl; 5 1st Chair and Department of Cardiology, Medical University of Warsaw, 02-091 Warsaw, Poland; krzysztof.filipiak@wum.edu.pl (K.J.F.); aleksandra.gasecka@wum.edu.pl (A.G.); 6 Maria Sklodowska-Curie Medical Academy in Warsaw, 02-034 Warsaw, Poland; maciej.cyran@uczelniamedyczna.com.pl; 7 Department of Emergency Medical Service, Wroclaw Medical University, 61-616 Wroclaw, Poland; 8 Department of Cardiology, University Medical Center Utrecht, 3584 Utrecht, The Netherlands; 9 Departments of General Anesthesiology and Outcomes Research, Anesthesiology Institute, Cleveland Clinic, Cleveland, OH 44195, USA; kurt.ruetzler@reflex.at; 10 Maria Sklodowska-Curie Bialystok Oncology Center, 02-034 Bialystok, Poland

**Keywords:** therapeutic hypothermia, cooling, controlled normothermia, post-resuscitation care, pediatric, outcome, meta-analysis

## Abstract

Cardiac arrest (CA) is associated with high mortality and poor life quality. Targeted temperature management (TTM) or therapeutic hypothermia is a therapy increasing the survival of adult patients after CA. The study aim was to assess the feasibility of therapeutic hypothermia after pediatric CA. We performed a systematic review and meta-analysis of randomized controlled trials and observational studies evaluating the use of TTM after pediatric CA. The primary outcome was survival to hospital discharge or 30-day survival. Secondary outcomes included a one-year survival rate, survival with a Vineland adaptive behavior scale (VABS-II) score ≥ 70, and occurrence of adverse events. Ten articles (*n* = 2002 patients) were included, comparing TTM patients (*n* = 638) with controls (*n* = 1364). In a fixed-effects meta-analysis, survival to hospital discharge in the TTM group was 49.7%, which was higher than in the non-TTM group (43.5%; odds ratio, OR = 1.22; 95% confidence interval, CI: 1.00, 1.50; *p* = 0.06). There were no differences in the one-year survival rate or the occurrence of adverse events between the TTM and non-TTM groups. Altogether, the use of TTM was associated with a higher survival to hospital discharge; however, it did not significantly increase the annual survival. Additional high-quality prospective studies are necessary to confer additional TTM benefits.

## 1. Introduction

Pediatric cardiac arrest (CA) is a sudden and devastating event with a low survival rate. Specifically for out-of-hospital CA (OHCA), survival to hospital discharge rates range from 2 to 27% [1,2,3]. Even fewer victims survive in a good neurological status (approximately 24–50% of the survivors [1,2]), with the rest experiencing long-term physical and psychological burdens, adversely affecting the quality of life [1,2].

This highly unsatisfactory outcome has pushed professionals to seek new treatments and refine the existing protocols in the hope for an improvement. In the recent decades especially, high expectations were associated with targeted temperature management (TTM), also known as mild therapeutic hypothermia. Much effort has been made globally to research these treatments and refine them with various techniques to achieve the goal [4]. After the return of spontaneous circulation, cooling can be applied externally (water, fanning, ice padding, blankets, caps) and/or internally (gastric lavage, bladder cooling, intravascular cooling via a catheter, trans-nasal evaporative cooling) [5,6]. None of the methods has shown a clear edge, although some have specific advantages, the discussion of which remains beyond the scope of this paper. There is no uniform globally accepted protocol, but it is generally agreed upon that the target temperature for TTM is 32–34 °C, with a minimum application time of 12 h [7,8].

This strategy has proven useful in two major trials in terms of survival and neurological outcome in adults suffering from CA, and since then it has been recommended by the 2013 International Liaison Committee on Resuscitation and 2010 American Heart Association guidelines [9,10]. More recently, it was found that there was no benefit of cooling to 33 °C when compared with less aggressive cooling only to a near-normal temperature of 36 °C, so-called controlled normothermia [11]. It appears that cooling is effective because it prevents fever, a common complication seen after CA [11]. The most recent HYPERION multicenter randomized controlled trial (RCT) provides further evidence on the beneficial role of TTM in preserving favorable neurological outcomes [12].

For newborn infants affected by perinatal hypoxia-ischemia, hypoxic ischemic encephalopathy, or birth asphyxia and suffering from neonatal encephalopathy, TTM has been demonstrated to improve outcomes. Several studies in neonates with asphyxia [13,14,15,16], all summarized in a 2013 Cochrane review, found that TTM was useful in full-term babies with encephalopathy [17]. Whole-body or selective head cooling to 33–34 °C, begun within six hours of birth and continued for 72 h, reduced the mortality, cerebral palsy, and neurological deficits in survivors.

On the basis of these adult and neonatal studies, it has also been recommended to use TTM as part of post-resuscitation management in children, a sheer extrapolation in the hope that these results are generalizable to pediatric populations [18]. However, large studies, like the recent in-hospital Therapeutic Hypothermia after Pediatric Cardiac Arrest (THAPCA-IN) and a similar out-of-hospital Therapeutic Hypothermia after Pediatric Cardiac Arrest (THAPCA-OH) trials, showed no clear benefit [19,20] either separately or in the pooled data analysis. The results revealed that in children after CA, cooling did not appear as useful as in adults [21]. Consequently, it has been argued by some that the THAPCA-OH study may prove a worthwhile clinical effect that is just too small to be picked up by the conventional statistical framework [22]. Unfortunately, neither survival nor any other secondary outcome measure were significantly different between the groups in this study.

Mounting data from the current literature and mounting doubts on the benefits of TTM have led us to carry out a systematic literature review and meta-analysis in this vital matter in order to determine whether there is an association between TTM and improved outcomes in pediatric patients after CA.

## 2. Materials and Methods

The present systematic review and meta-analysis followed the PRISMA statement for reporting systematic reviews and meta-analyses [23] and the MOOSE statement recommendations for reporting systematic reviews and meta-analyses of observational studies [24]. Because of its nature, the study did not need to be approved by an institutional review board.

### 2.1. Search Strategy

Publications were identified via systematic searches of Ovid Embase, Ovid Medline, Web of Science, the Cochrane Central Register of Controlled Trials, and the publisher subset of PubMed from inception to 20 October 2020.

The search was performed by using the following terms: “targeted temperature management” OR “TTM” OR “hypothermia” OR “therapeutic hypothermia” OR “mild hypothermia” AND “cardiac arrest” OR “CA” OR “heart arrest” OR “circulation arrest” OR “cardiopulmonary resuscitation” OR “CPR” OR “OHCA” OR “IHCA” OR “return of spontaneous circulation” OR “ROSC” OR “cardiac ventric* fibrillation” OR “heart ventric* fibrillation” OR “pulseless ventric* tachycardia” OR “asysto*” OR “pulseless electrical activity” AND “pediatric” OR “child” OR “infant”. No language, publication date, or publication status restrictions were applied. Additionally, all of the references listed in the identified articles were reviewed, and manual searching for related articles was conducted in order to recognize all eligible studies and achieve minimal publication bias. The bibliographic records retrieved were downloaded, imported, and de-duplicated in the EndNote software.

### 2.2. Selection Criteria

Studies included in this meta-analysis fulfilled the following criteria (PICOS): (1) participants: patients with CA due to any cause under 18 years old; (2) intervention: TTM; (3) comparison: standard care; (4) outcomes: detailed information on survival; (5) study design: RCT, quasi-randomized, or observational study comparing TTM and standard care for their effects in patients with CA.

Studies were excluded if they were reviews, animal studies, case reports, letters, conference or poster abstracts, or articles not containing original data.

### 2.3. Study Selection

The studies were independently screened by two authors (W.W. and L.S.), verifying the titles and abstracts for potential eligibility. Secondly, after reviewing full texts, the authors included eligible studies in accordance with the previously assumed inclusion criteria. Discrepancies regarding the selection of articles were resolved by consensus with a third reviewer (J.S.).

### 2.4. Data Extraction

Two authors (W.W. and A.G.) independently extracted and recorded the desirable information of each enrolled study, which consisted of the study title, first author, relevant demographic data, intervention and control, results for outcomes, inclusion and exclusion criteria, outcome definitions, and findings. For any missing information, we attempted to contact the corresponding authors by email for full original data. A third author (L.S.) was consulted in cases of disagreement between the two authors.

### 2.5. Outcomes

The primary outcome of the current meta-analysis was survival to hospital discharge or 30-day survival. The secondary outcomes were adverse events and rates of other survival periods.

### 2.6. Quality Assessment

The quality assessment of all retained articles was performed by two independent reviewers (W.W. and J.S.). Discrepancies regarding the quality of articles were resolved by consensus with a third reviewer (L.S.). The ROBINS-I tool (serving to assess the risk of bias in non-randomized studies of interventions) was used to evaluate the quality of non-randomized trials [25], and the RoB 2 tool (revised tool for determining the risk of bias in randomized trials) was applied to assess the quality of randomized studies [26]. The robvis application served to visualize the risk of bias assessments [27]. The scale has seven main domains (confounding, participant selection, classification of interventions, deviation from interventions, missing data, outcome measurement, and selection of reported results), and assigns one point for each of the following three judgements: critical, moderate, and low. The review authors’ judgments about each risk of bias item are provided in Supplementary Figures S7–S10. Additionally, we applied the Grading of Recommendations Assessment, Development and Evaluation (GRADE) approach [28] with GRADEpro software (available online http://gradepro.org, accessed on 3 March 2021) to assess the quality of evidence of the main outcomes (Table S4).

### 2.7. Statistical Analysis

All statistical analyses were performed with Review Manager software 5.4 (The Cochrane Collaboration, Copenhagen, Denmark). Outcomes were summarized by using the Mantel–Haenszel risk ratios or mean differences. All results are presented with their 95% confidence intervals (CIs). When a continuous outcome was reported in a study as the median, range, and interquartile range, we estimated means and standard deviations using the formula described by Hozo et al. [29]. Heterogeneity was assessed statistically with I^2^ (no heterogeneity: I^2^ of 0–25%; moderate heterogeneity: I^2^ of  25–50%; large heterogeneity: I^2^ of 50–75%; extreme heterogeneity: I^2^ of 75–100%) [30]. The random effects model was used for I^2^ > 50%; otherwise, the fixed effects model was employed. The value of *p* < 0.05 was assumed to indicate statistical significance. The statistical testing was two-tailed.

We looked for a potential publication bias by using a funnel plot if more than 10 trials were included for an outcome. For continuous outcomes, the Egger test was used to detect funnel plot asymmetry [31]. For dichotomous outcomes, we applied the arcsine test. All analyses were performed with the Review Manager or Statistica 13.4EN software.

GraphPad Prism 8 served to create the survival curve of pooled analysis of randomized trials (GraphPad Software, San Diego, CA, USA).

## 3. Results

### 3.1. Study Selection

**Figure 1:**
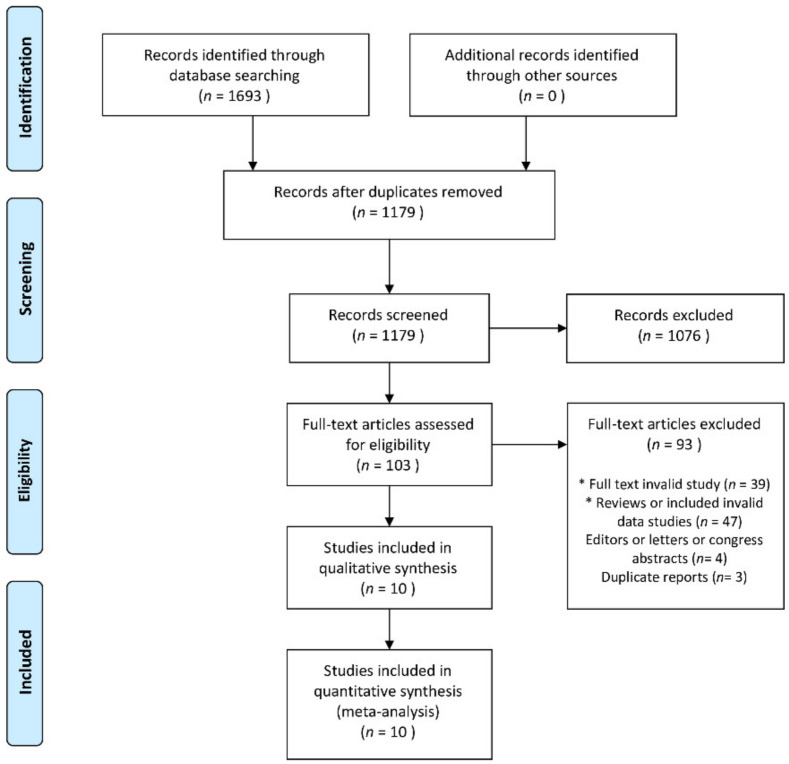
Flow diagram showing the stages of database searching and study selection per the PRISMA guidelines.

A total of 1693 records were identified after the initial research. After eliminating duplicate citations and studies that did not meet the eligibility criteria, 103 full-text articles were retrieved for complete review (Figure 1). Ninety-three studies were subsequently excluded, leaving 10 articles included in the review, with a combined total of 2002 individual patients: 638 in the TTM group and 1364 in the non-TTM group.

### 3.2. Study Characteristics

The characteristics of the 10 included studies are presented in Table 1, Tables S1 and S2. The author, year and country of publication, study type, and the participants’ number, age, and gender are presented for the TTM and non-TTM groups [19,20,21,32,33,34,35,36,37,38].

Of the 10 studies, two were RCTs [19,20]. The study sample size ranged from 43 to 663 patients. Nine were single-country trials, and one was a multi-country trial. Of the single-country trials, two were conducted in the USA [33,35], two in Taiwan [36,37], one in Korea [31], one in the UK [21], and one in the Netherlands [38]. The multicenter studies were mostly conducted in the USA and Canada [20] and the UK and Canada [34], and one was performed in the USA, one in Canada, and one in the UK [19]. Detailed patient characteristics are presented in the Supplementary Material (SM, Figures S1–S6). The results for the quality of evidence are summarized in Supplementary Figures S7–S10.

### 3.3. Primary Outcome

**Figure 2:**
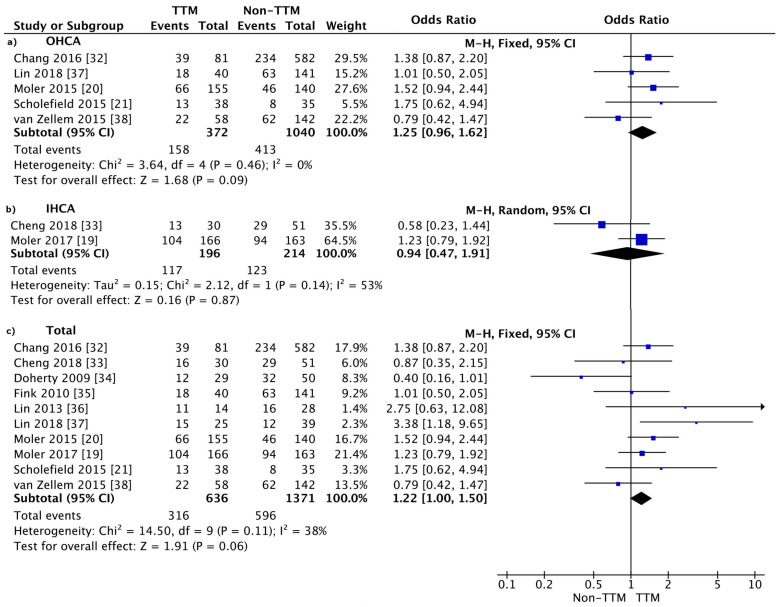
Forest plot of survival to hospital discharge in the TTM and non-TTM groups: (**a**) in OHCA; (**b**) in IHCA; and (**c**) in total. The center of each square represents the weighted odds ratios for individual trials, and the corresponding horizontal line stands for the 95% CI. The diamonds represent pooled results. CI = confidence interval; IHCA = in-hospital cardiac arrest; OHCA = out-of-hospital cardiac arrest; TTM = targeted temperature management.

Ten studies indicated that the survival to hospital discharge or 30-day survival parameters [19,20,21,32,33,34,35,36,37,38]. Patient survival in the TTM group was 49.7%, and turned out slightly higher than that in the non-TTM group (43.5%; odds ratio (OR) = 1.22; 95% CI: 1.00, 1.50; *p* = 0.06; I^2^ = 38%; Figure 2). The calculated Cohen’s *h* effect size estimate was 0.12. By the common convention, this represents less than a small effect size.

The sub-analysis showed that the survival to hospital discharge or 30-day survival rate was higher in the TTM compared with the non-TTM group for OHCA (42.5% vs. 39.7%; OR = 1.25; 95% CI: 0.96, 1.63; *p* = 0.09; I^2^ = 0%), as well as for in-hospital cardiac arrest (IHCA) (59.7% vs. 57.5%; OR = 0.94; 95% CI: 0.47, 1.91; *p* = 0.87; I^2^ = 53%).

The survival to hospital discharge or 30-day survival rate was reported in only one RCT in OHCA: the survival in the TTM vs. non-TTM groups varied, and amounted to 42.6% vs. 32.9% (OR = 1.52; 95% CI: 0.94, 2.44; *p* = 0.09). Moreover, one study reported survival after IHCA in the TTM vs. non-TTM groups (62.7% vs. 57.7%; OR = 1.92; 95% CI: 0.79, 1.92; *p* = 0.36). In none of the cases was the difference statistically significant.

### 3.4. Secondary Outcomes

**Figure 3:**
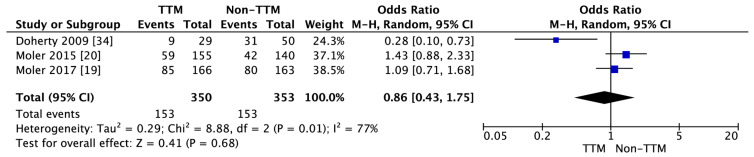
Forest plot of the survival rate in a six-month follow-up in the TTM and non-TTM groups. The center of each square represents the weighted odds ratios for individual trials, and the corresponding horizontal line stands for the 95% CI. The diamonds represent pooled results. CI = confidence interval; TTM = targeted temperature management.

Three studies indicated survival at a six-month follow-up [19,20,34]. The survival of patients in the TTM group and the control group equaled 43.7% and 43.3%, respectively (OR = 0.86; 95% CI: 0.43, 1.75; *p* = 0.68; I^2^ = 77%; Figure 3).

Subgroup analysis revealed that only one RCT reported survival at a six-month follow-up regarding OHCA [20], and one regarding IHCA [19]. The survival rate in a six-month follow-up was higher in the TTM group compared with the non-TTM group in OHCA (38.1% vs. 30.0%; OR = 1.43; 95% CI: 0.88, 2.33; *p* = 0.15) and IHCA (51.2% vs. 49.1%; OR = 1.09; 95% CI: 0.71, 1.68; *p* = 0.70).

**Figure 4:**
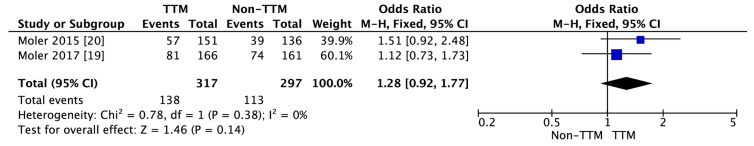
Forest plot of the survival rate in a one-year follow-up in the TTM and non-TTM groups. The center of each square represents the weighted odds ratios for individual trials, and the corresponding horizontal line stands for the 95% CI. The diamonds represent pooled results. CI = confidence interval; TTM = targeted temperature management

**Figure 5:**
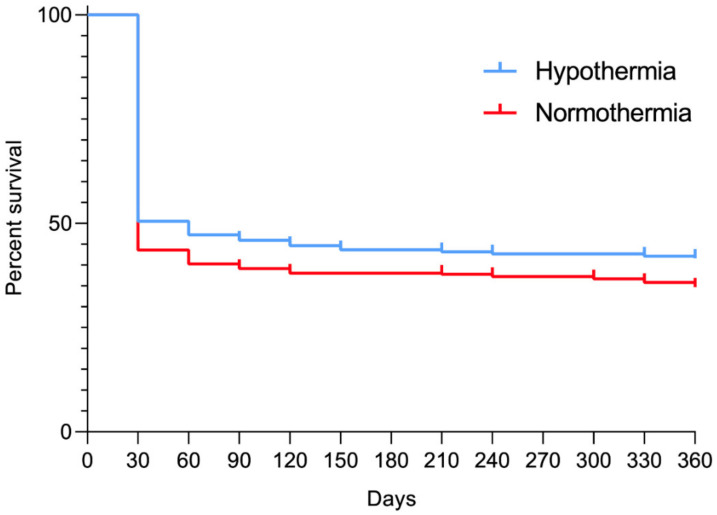
Pooled analysis of survival from 0 to 360 days after cardiac arrest.

The one-year survival rate was demonstrated in two studies [19,20], and equaled 43.5% for patients treated with TTM and 38.0% for the non-TTM group (OR = 1.28; 95% CI: 0.92, 1.77; *p* = 0.14; I^2^ = 0%; Figure 4) [18,33,34,35]. Additionally, a pooled data analysis in the survival curve context was performed. The results are presented in Figure 5.

Only one RCT reported the one-year survival rate after OHCA, and one after IHCA. The one-year survival rate in the TTM group compared with the non-TTM group was 37.7% vs. 28.7% (OR = 1.51; 95% CI: 0.92, 2.48; *p* = 0.10) for OHCA, and 48.8% vs. 46.0% (OR = 1.12; 95% CI: 0.73, 1.73; *p* = 0.61) for IHCA.

**Figure 6:**
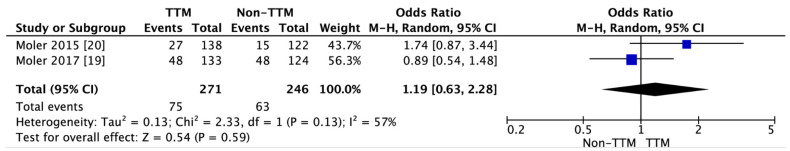
Forest plot of the survival with a VABS-II score ≥ 70 points at a one-year follow-up in the TTM and non-TTM groups. The center of each square represents the weighted odds ratios for individual trials, and the corresponding horizontal line stands for the 95% CI. The diamonds represent pooled results. CI = confidence interval; TTM = targeted temperature management.

Two studies presented survival in the Vineland Adaptive Behavior Scale (VABS)-II score ≥ 70 at the one-year follow-up [19,20]. In the pooled analysis, 27.7% of the patients treated with TTM survived one year, with a VABS-II score ≥ 70 points, compared with 25.6% in the non-TTM group (OR = 1.19; 95% CI: 0.63, 2.28; *p* = 0.59; I^2^ = 57%; Figure 6).

TTM was used more frequently for shockable rhythms compared with therapy without TTM (12.3% vs. 8.6%; OR = 1.54; 95% CI: 0.81, 2.92; *p* = 0.19; I^2^ = 64%). For non-shockable rhythms, the opposite trend was observed (26.2% vs. 26.4%; OR = 0.94; 95% CI: 0.76, 1.17; *p* = 0.60; I^2^ = 0%; SM).

The statistical analysis showed no statistically significant differences in the occurrence of adverse events in the TTM vs. non-TTM groups. A summary of adverse event occurrence is presented in the Table S3.

## 4. Discussion

Pediatric CA is a rare but sudden and devastating event, with a disappointingly low survival rate. TTM has proven to be useful in adult CA and neonatal asphyxia. Conflicting results provided in the recent literature on TTM use in children have led to this meta-analysis, aiming to summarize the state-of-the-art material in the subject matter.

Ten papers were found eligible, with only two representing RCTs. The total number of patients equaled 2002: 638 in the TTM group and 1364 in the control (non-TTM) group. All studies provided data on the primary outcome. Only one study favored intervention (TTM), one favored the control, and the rest were inconclusive. On the basis of the meta-analysis, the study found a statistically non-significant difference in 30-day survival (49.7% vs. 43.5%, respectively; OR = 1.22 (1.00–1.50); *p* = 0.06). With the current body of scientific evidence and within the conventional statistical framework, there is no proof of TTM superiority over the control. The sample size of this meta-analysis is decent, so it is also not a matter of insignificant statistical power. The problem seems to lie in the clinical effect size. The calculated effect size was *h* = 0.11, i.e., less than small by Cohen’s convention. Furthermore, the sub-analysis of IHCA and OHCA revealed no statistical evidence to favor one of the groups. Vincent and Taccone made their point in showing the parents’ perspective of seeking hope in despair [22] that clinicians readily like to share, regardless of the scientific evidence. With one or two more studies, the next meta-analysis may reach statistical significance, but by no means will this change the effect size.

Longer-term survival observations were unfortunately also disappointing. The survival to discharge at a six-month follow-up did not differ between the groups, as evidenced by three studies (43.7% vs. 43.3%; OR = 0.86 (0.43–1.75)). Subgroup analysis (IHCA and OHCA) based on one RCT each showed no difference.

Similarly, the one-year survival rate based on two studies was not favorable for TTM (43.5% vs. 38.7%; OR = 1.28 (0.92–1.77)). The one-year neurological outcomes were not different between the two groups, with a VABS-II score ≥ 70 in 27.7% vs. 25.6% (OR = 1.19 (0.63–2.28)) in the TTM vs. control group, as based on three studies. Finally, subgroup analysis was based on the initial heart rhythm, as it is considered to be a significant prognostic factor for the outcome. We have found no difference in 30-day survival between the TTM and control group in the case of initially shockable and non-shockable rhythm. Importantly, there was no statistically significant difference in the number of adverse events between the studied groups.

Short-term survival, preferably with a favorable neurological outcome, is a key to further recovery. TTM is a treatment intervention aimed at long-term survival and satisfactory quality of life. It is unclear from the literature at hand why the TTM group, doing slightly (but statistically insignificantly) better at 30 days, turns out not better at one year. Possibly, there is much in the patient care to be improved in the post-discharge period. This should include optimizing the diagnosis and treatment of diseases that are the underlying causes of CA, as well as home monitoring with telehealth technologies, all in an effort to prevent secondary CA. Furthermore, cardiopulmonary and neurological rehabilitation programs, even if available, suffer practical limitations.

Considering the equivocal results, as well as the additional equipment costs and personnel burden of the studied intervention, a separate cost efficiency analysis is warranted.

It seems that there is a lingering positive effect of TTM that we may be somehow missing [22,39]. Currently, TTM should still be considered as an experimental procedure, with a potentially favorable risk to benefit ratio, but without fully proven efficacy. Most of the studies at hand were based on a mixed pediatric population with various underlying morbidities and causes leading to CA. Thus, better planned, multicenter RCTs focusing on well-defined, homogenous cohorts with long-term follow-up are necessary. It is conceivable that these future studies will tease out subpopulations benefiting from the treatment discussed. Finally, since it is believed that there is a dose–effect relationship of TTM on neuroprotection, a careful reevaluation seems necessary of the TTM protocol in terms of cooling methods, target temperature, time-domain parameters (time to TTM start, time to target temperature, total TTM duration), and the rewarming procedure [40,41].

The experience so far has proven that this is notoriously difficult. Even in the most recent HYPERION study, although well-designed with stringent inclusion criteria, there were several potentially important differences in the patients’ baseline characteristics, and the fragility index was 1, both undermining the positive primary finding [12].

### Limitations

Many of the studies included have their flaws. Most importantly, only two were RCTs, with the remaining studies having a retrospective observational design. This has important implications, possibly leading to an allocation bias or other types of selection bias. It has been previously shown that allocation bias can cause up to 30–40% of the shift in treatment effect estimation in both directions [41]. As noted by Lewis et al. in a TTM for head trauma meta-analysis, studies with properly concealed allocation showed no effect of the intervention, while those with no or unclear concealment demonstrated a statistically significant treatment benefit [42].

Other significant downsides of the available evidence are the small sample size (48 at the lowest end) [36], mixed pediatric populations, including a broad age range (from infants to 21-year-olds), different settings of and mechanisms leading to CA (OHCA vs. IHCA), various causes of CA (cardiac, non-cardiac, trauma, etc.), and various baseline morbidities and comorbidities, all being a consequence of heterogenous inclusion and exclusion criteria in the available studies. These factors may opacify the view on the benefiting subpopulations. It is perfectly conceivable that the TTM effect on children depends on the primary diagnosis and overall prognosis.

Another considerable limitation is the non-uniformity of the applied protocols, most importantly, the target temperature applied ranging from 32 °C to 35 °C, but also the time domain parameters and the rewarming procedure (as specified in detail in the SM). Moreover, frequent deviations from the protocol were reported in some studies [33].

This inconsistency in the input studies may have diluted the positive effect of the TTM. On the other hand, any strong benefit should have already stood out clearly with the decent sample size of this meta-analysis.

## 5. Conclusions

In summary, on the basis of the pooled results of the available literature, this meta-analysis succeeded in showing a clinically minor but statistically significant effect of TTM on the primary outcome, i.e., 30-day survival, but not on any other studied endpoint. Specifically, this meta-analysis did not demonstrate any significant increase in the annual survival or neurological status. The lack of evidence for a long-term survival benefit could be due to a small number of studies reporting long-term outcomes, and therefore a smaller number of patients included in the meta-analysis for these measures. The main implication of this analysis is that better-planned, high-quality multicenter RCTs with more homogenous populations and a long-term follow-up are necessary.

Considering the equivocal results, as well as the additional equipment costs and personnel burden of the investigated intervention, a separate cost efficiency study is warranted. It seems that there is a lingering positive effect of TTM that we somehow miss. Currently, TTM should be still considered and applied in strictly controlled experimental settings, preferably as well-planned, high-quality multicenter RCTs with a long-term follow-up. Further studies are necessary, because most research at hand was based on a mixed pediatric population with various underlying morbidities and causes leading to CA. It is conceivable that these future studies will tease out subpopulations benefiting from the treatment discussed. Finally, since it is believed that there is a dose–effect relationship of TTM on neuroprotection, a careful reevaluation of the TTM protocol seems necessary in terms of cooling methods, target temperature, time domain parameters (time to TTM start, time to target temperature, total TTM duration), and the rewarming procedure.

## Figures and Tables

**Table 1:** Characteristics of included studies.

Study	Country	Study Design	Cardiac Arrest Setting	TTM Group			Non-TTM Group		
				No.	Age, Years	Males, *n* (%)	No.	Age, Years	Males, *n* (%)
Chang et al., 2016	Korea	Cross-sectional observational	OHCA	81	14.5 ± 1.3	25 (30.9)	582	7.5 ± 2.3	199 (34.2)
Cheng et al., 2018	USA	Retrospective cohort	IHCA	26	0.8 ± 0.6	12 (46.2)	49	0.4 ± 0.3	33 (67.3)
Doherty et al., 2009	Canada/UK	Retrospective observational multicenter	OHCA and IHCA	29	NR	16 (55.2)	50	NR	23 (46.0)
Fink et al., 2010	USA	Retrospective cohort	OHCA and IHCA	40	6.0 ± 6.6	24 (60.0)	141	6.0 ± 6.2	80 (56.7)
Lin et al., 2013	Taiwan	Retrospective cohort	OHCA and IHCA	15	NR	10 (66.7)	28	NR	18 (64.3)
Lin et al., 2018	Taiwan	Retrospective cohort	OHCA	25	NR	21 (84.0)	39	NR	28 (71.8)
Moler et al., 2015	USA/Canada	RCT	OHCA	155	3.7 ± 1.6	102 (65.8)	140	2.7 ± 1.1	73 (52.1)
Moler et al., 2017	USA/Canada/UK	RCT	IHCA	166	2.2 ± 0.9	97 (58.4)	163	4.3 ± 1.8	99 (60.7)
Scholefield et al., 2015	UK	Retrospective cohort	OHCA	38	2.2 ± 1.7	17 (44.7)	35	1.5 ± 1.2	8 (22.9)
Van Zellem et al., 2015	The Netherlands	Observational cohort	OHCA	63	6.5 ± 5.1	43 (68.3)	137	6.4 ± 6.3	67 (48.9)

Legend: IHCA = in-hospital cardiac arrest; NR = not reported; OHCA = out-of-hospital cardiac arrest; RCT = randomized controlled trial; TTM = targeted temperature management.

## Data Availability

Not applicable.

## Supplementary Materials

The following are available online at https://www.mdpi.com/article/10.3390/jcm10071389/s1: Table S1: Inclusion and exclusion criteria of the analyzed studies; Table S2: Targeted temperature management (TTM) and non-TTM definitions in the included studies; Table S3: Adverse events; Table S4: Cardiac etiology of cardiac arrest in the TTM and non-TTM groups; Table S5. The Grading of Recommendations Assessment, Development and Evolution (GRADE) approach; Figure S1: Forest plot of patients’ age in the TTM and non-TTM groups; Figure S2: Forest plot of patients’ gender (male) in the TTM and non-TTM groups; Figure S3: Forest plot of cardiac etiology of cardiac arrest in the TTM and non-TTM groups; Figure S4: Forest plot of witnessed cardiac arrest in the TTM and non-TTM groups; Figure S5: Forest plot of bystander cardiopulmonary resuscitation in the TTM and non-TTM groups; Figure S6: Funnel plot of the odds ratio (OR) with standard error: (A) survival to hospital discharge, (B) survival rate in a six-month follow-up, (C) survival rate in a one-year follow-up, (D) survival with a VABS-II score ≥ 70 points in a one-year follow-up; Figure S7: A summary table of the review authors’ judgements for each risk of bias item for particular randomized studies. Figure S8: A plot of the distribution of the review authors’ judgements across randomized studies for each risk of bias item. Figure S9: A summary table of the review authors’ judgements for each risk of bias item for particular non-randomized studies. Figure S10: A plot of the distribution of the review authors’ judgements across non-randomized studies for each risk of bias item.
