# Supplementary material for: Efficacy of Targeted Temperature Management after Pediatric Cardiac Arrest: A Meta-Analysis of 2002 Patients
Source: J Clin Med. 2021 Mar 30;10(7):1389. doi: 10.3390/jcm10071389 (PMC8037776; doi:10.3390/jcm10071389)
Supplement: Supplementary file 1 [file jcm-10-01389-s001.pdf]

Journal of
Clinical Medicine

MDPI

Efficacy of Targeted Temperature Management After Pediatric Cardiac Arrest: A Meta-Analysis of 2002 Patients

Supplementary material

Table S1. Inclusion and exclusion criteria of included studies.

Study	Inclusion Criteria	Exclusion Criteria	Outcome(s)	Colling Method	Rewarming Setting	Findings
Chang et al. 2016	EMS-assessed OHCA patients who survived to admission and were 18 years of age or younger, regardless of the cause (presumed cardiac or non-cardiac origin)	(1) unknown neurological status at hospital discharge; (2) an alert mental status after resuscitation at the ED.	Survival to discharge. Good neurological status, defined as having a Cerebral Performance Category (CPC) score of 1 (good performance, no neurological disability) or 2 (moderate disability, can work).	Core temperature 32–34◦C for at least 12h	NS	MTH and the effect of MTH across the initial ECG at the scene were not significantly associated with survival or good neurologic recovery in pediatric OHCA survivors.
Cheng et al. 2018	Patients treated under the TH protocol in the first 18 months of its initiation	Known significant intracranial hemorrhage (large epidural, subdural, or parenchymal hemorrhage, or Grade III or IV intraventricular hemorrhage), pregnancy, or postmenstrual age < 36 weeks.	Overall neurologic outcome.	33.5°C for either 72h (<1year of age) or 48h (1year of age). Patients < 1 year of age were cooled for 72 h based on published neo- natal trials, while patients 1 year of age were cooled 48 h	0.5 °C every 2 h to a goal temperature of 36.5 °C	Pediatric CHD patients who suffer cardiac arrest can be treated effectively and safely with TH, which may decrease the incidence of seizures.
Doherty et al. 2009	Patients >40 weeks postconceptual age	Adequate data could not be extracted	(1) Mortality at 6 months; (2) Pediatric	Temperature of <35°C within 6 hours	NS	Hypothermia therapy was used in

	and <18 years of age; cardiac arrest of at least 3 minutes duration; survival for at least 12 hours after return of spontaneous circulation (or the commencement of rescue ECMO flow); and admission to the intensive care unit after resuscitation	from the chart. neonates admitted to the neonatal intensive care unit directly from the delivery room with a diagnosis of birth asphyxia, because this population has been studied previously.	Cerebral Performance Category (PCPC) score, assessed at 6 months after cardiac arrest; (3) when the PCPC assessment was possible before cardiac arrest, the ΔPCPC at 6 months was calculated; (4) duration of mechanical ventilation; (5) length of stay in the intensive care unit; (6) length of stay at an acute-care hospital (tertiary care facility); (7) multiorgan dysfunction scores and pediatric logistic organ dysfunction scores for 3 days; and (8) data on hypothermia-related adverse events (eg, infections, bleeding or thrombosis, and arrhythmias), recorded for 14 days after cardiac arrest.	of cardiac arrest for a continuous period of at least 12 hour.		resuscitation scenarios that are associated with greater risk of poor outcome. In an adjusted analysis, the effectiveness of hypothermia therapy was neither supported nor refuted.
Fink et al. 2010	Infants and children 1 wk to 21 yrs of age admitted to the ICU with ROSC after CA. CA was defined as receipt of chest compressions for pulselessness as	Children with congenital heart disease	Logistics related to the application of HT and the frequency of adverse events in the first 4 days after CA. Adverse events included	The median therapeutic hypothermia target temperature was 34.0°C (33.5–34.8°C), was reached by 7 hrs (5–8 hrs) after admission in patients who were not	Re-warming was achieved by increasing the set point of the cooling blanket gradually until the patient reached 36°C, at which time the blanket was	Therapeutic hypothermia was feasible, with target temperature achieved in <3 hrs overall. Temperature below target range

	determined by a health care worker		hemorrhage, transfusion, positive cultures, electrolyte supplementation, intermittent arrhythmia and rearrest. Secondary outcomes included mortality and Glasgow Outcome Score (GOS) (assigned in a non-blinded fashion based on medical records for both pre-arrest and hospital discharge status), a variation on the Utstein recommendation.	hypothermic on admission and was maintained for 24 hrs (16–48 hrs).	turned off to prevent over-warming.	was associated with increased mortality.
Lin et al. 2013	Age from 2 months to 18 years; cardiac arrest of at least 3 minutes' duration; survival for at least 12 hours after return of spontaneous circulation; and admission to the intensive care unit after resuscitation	Children with congenital heart diseases. If a patient experienced more than one resuscitation during the study period, only the first resuscitation meeting the eligibility criteria was included.	The primary outcomes included survival and Pediatric Cerebral Performance Category (PCPC) scores at hospital discharge. The secondary outcomes were related to the application of therapeutic hypothermia and the hypothermia-related adverse events.	Temperature of 33°C within 6 hours of cardiac arrest for a continuous period of 24 or 72 hours	Rewarming was achieved by increasing the set point of the temperature management system gradually at 1°C per day until the patient reached 36°C	Therapeutic hypothermia was associated with increased survival rate after pediatric resuscitation.
Lin et al. 2018	1) age from 1 month to 18 years; 2) duration of cardiac arrest at least 3 min and ROSC after resuscitation; 3) comatose status (Glasgow	1) who were older than 18 years; 2) with hemodynamic instability refractory to intensive care and who died within 12 h; 3) who were not	The primary outcome was neurological outcome, which was assessed using PCPC. The secondary outcomes were survival rate at 30	Target temperature of 33 °C, cooling duration of the maintenance phase of 72 h for the children with asphyxial etiology.	Rewarming was achieved by gradually increasing the temperature on the management system by 1 °C per day until	Paediatric asphyxial OHCA was associated with high mortality and morbidity. Seventy-two-hour therapeutic

	Coma Scale (GCS) score ≤ 8) after ROSC; and 4) survival for 12 h or more after the return of circulation	in a coma after resuscitation (GCS > 8); 4) known to have pre-existing degenerative neurological diseases; 5) with traumatic brain injury; and 6) with ventricular fibrillation and a history of congenital heart disease.	days following cardiac arrest, duration of hospitalization, and the prevalence of therapeutic hypothermia-related adverse events. Adverse events included hemodynamic changes, intermittent arrhythmia, coagulopathy, electrolyte imbalance and infection during hypothermia, and rebounding increased intracranial pressure and seizures after rewarming.		the patient reached 36 °C.	hypothermia was associated with a better 1-month survival rate and 6-month neurological outcomes than normothermia in our pediatric patients with asphyxial OHCA.
Moler et al. 2015	Children older than 48 hours and younger than 18 years of age were eligible for inclusion in the study if they had a cardiac arrest requiring chest compressions for at least 2 minutes and remained dependent on mechanical ventilation after the return of circulation.	The inability to undergo randomization for any reason within 6 hours after the return of circulation, a score of 5 or 6 on the Glasgow Coma Scale motor-response subscale (on which scores range from 1 to 6, with lower scores indicating reduced levels of function), the decision by the clinical team to withhold aggressive treatment, and major trauma associated with the cardiac arrest.	The primary outcome was survival with a good neurobehavioral outcome at 12 months of follow-up. Secondary outcomes were survival 12 months after cardiac arrest and change in neurobehavioral function, measured as the difference be- tween the baseline level before cardiac arrest and the 12-month measurement on the VABS-II.	Core temperature of 33.0°C (range, 32.0 to 34.0) for 48 hours.	The children were then rewarmed over a period of 16 hours or longer to a target temperature of 36.8°C (range, 36.0 to 37.5).	In comatose children who survived out-of-hospital cardiac arrest, therapeutic hypothermia, as compared with therapeutic normothermia, did not confer a significant benefit in survival with a good functional outcome at 1 year

Moler et al. 2017	Children older than 48 hours and younger than 18 years of age were eligible for inclusion if they had a cardiac arrest that began within the walls of a hospital, received chest compressions for at least 2 minutes, and remained dependent on mechanical ventilation after the return of circulation.	A score of 5 or 6 on the Glasgow Coma Scale motor-response subscale (on which scores range from 1 to 6, with lower scores indicating worse function), the inability to undergo randomization within 6 hours after the return of circulation, active and refractory severe bleeding, a preexisting illness associated with a life expectancy of less than 12 months, and a decision by the clinical team to withhold aggressive treatment.	The primary outcome was survival with a favorable neurobehavioral outcome at 12 months of follow-up. Secondary outcomes were survival at 12 months after cardiac arrest and change in neurobehavioral function, which was measured as the difference between the baseline measurement (before cardiac arrest) and the 12-month measurement on the VABS-II.	core temperature of 33.0°C (range, 32.0 to 34.0) for 48 hours.	The patients were then rewarmed over a period of 16 hours or longer to a target temperature of 36.8°C (range, 36.0 to 37.5)	Among comatose children who survived in-hospital cardiac arrest, therapeutic hypothermia, as compared with therapeutic normothermia, did not confer a significant benefit in survival with a favorable functional outcome at 1 year.
Scholefield et al. 2015	Aged between at least one day and 16 years, admitted to ICU after an OHCA with return of spontaneous circulation (ROSC).	NS	Primary outcome was survival to hospital discharge; efficacy and safety outcomes included: application of TTM, physiological, hematological and biochemical side effects. Secondly, the proportions with abnormal values or adverse events within 72 h of ICU admission were compared.	32–34◦C for 24 h	controlled rewarming, by 0.5 ◦ C every 2 h, and 37 ◦ C	TTM (32–34◦C) was feasible but associated with bradycardia, hypotension, and increased length of stay in ICU. Temperature <32 ◦ C had a universally grave prognosis. Larger studies are required to assess effect on survival.

van Zellem et al. 2015	Patients aged >28 days and <18 years with documented CA. (1) all children resuscitated in-hospital (e.g. emergency department, ward, or ICU) and out-of-hospital, and consecutively admitted to our ICU, and (2) children resuscitated in a regional hospital or other university hospital, and after ROSC consecutively admitted to our ICU.	Neonatal resuscitations, children with cyanotic congenital heart disease, and children without an arterial line.	The primary outcome measure was IH mortality. In the second analysis of the first research question, the "area under the curve" (AUC) of PaO_2 was calculated for each patient to determine the influence of the cumulative PaO_2 on in-hospital mortality.	Target temperature is 32–34 ◦ C for 24 h following ROSC.	Rewarmed passively at a rate of 0.5 ° C per 2 h.	Cumulative PaO_2 analysis showed that the IH mortality is significantly lower in MTH-treated children with high PaO2 levels.

Legend: AUC = area under the curve; CA = Cardiac arrest; CPC = Cerebral Performance Category; ICU = Intensive Care Unit; GCS = Glasgow Coma Scale; NS = Not specified; OHCA = Out-of-hospital cardiac arrest; ROSC = Return of spontaneous circulation; TTM = Targeted temperature management;.

Table S2. Targeted temperature management (TTM) and not-TTM definitions in included studies.

Study	Definition of Targeted Temperature Management	Definition of Non-Targeted Temperature Management
Chang et al. 2016	Case in which patients received therapeutic hypothermia (core temperature 32–34◦C) after recovering spontaneous circulation (ROSC) by using a method such as external cooling (water, fanning, or ice padding), internal cooling (gastric lavage, bladder cooling, or intravascular cooling using a catheter) or mixed cooling.	Normothermia.
Cheng et al. 2018	Temperature was targeted to be 33.5°C for either 72h (<1year of age) or 48h (1year of age). Patients < 1 year of age were cooled for 72 h based on published neo- natal trials, while patients 1 year of age were cooled 48 h. Cooling was managed via the ECMO circuit or via cooling blanket for those not on ECMO. Per protocol, patients were rewarmed at a rate of 0.5 °C every 2 h to a goal temperature of 36.5 °C, although goal temperature was set by the medical care team.	For control patients, data were collected over a "therapeutic window" time period which paralleled the data collection time period for TTM patients. The "therapeutic window" consisted of either 72 h (< 1 year of age) or 48 h (1 year of age) to represent the TTM time period post-arrest, plus 12 h to represent rewarming. Thus, control patients had temperature and monitoring data collected for 84 h post-arrest if < 1 year of age and 60 h if ≥1 year of age.

Doherty et al. 2009	all patients who were cooled to a temperature of ≤ 35°C within 6 hours of cardiac arrest for a continuous period of at least 12 hours	Normothermia.
Fink et al. 2010	Induction of HT was accomplished with multiple modalities. Most commonly, we used a cooling blanket (Cincinnati SubZero Plastipad, Cincinnati, OH) positioned under the patient and controlled by an automated cooling system (Gaymar Medi-Therm III, Orchard Park, NY) set to the target temperature. Other methods included surface cooling with ice packets, bath and fan, lowering of the room and ventilator humidifier thermostat, and, occasionally, gastric lavage with iced saline. One patient received 40 mL/kg of intravenous iced saline to induce HT. Re-warming was achieved by increasing the set point of the cooling blanket gradually until the patient reached 36°C, at which time the blanket was turned off to prevent over-warming.	Normothermia.
Lin et al. 2013	Patients who were cooled to a temperature of 33°C within 6 hours of cardiac arrest for a continuous period of 24 or 72 hours. Induction of therapeutic hypothermia was accomplished with thermal heat-exchange cooling pads attached to the patient and controlled by an automated temperature management system (Arctic SunTM, Medivance, Inc.) set to the target temperature. Neuromuscular blockers were used to prevent shivering during induction of therapeutic hypothermia. Hyperthermia was prevented in both groups as recommended by the current International Liaison Committee on Resuscitation guidelines. Rewarming was achieved by increasing the set point of the temperature management system gradually at 1°C per day until the patient reached 36°C	Normothermia.
Lin et al. 2018	Induction of therapeutic hypothermia was accomplished with thermal heat- exchange cooling pads according to the patient's age and size, and controlled using an automated temperature management system (Arctic Sun, Medivance Inc. Louisville, CO, USA) set to a target temperature of 33 °C. From our past experience of critical care for neonatal asphyxia, we used a cooling duration of the maintenance phase of 72 h for the children with asphyxial aetiologies. Rewarming was achieved by gradually increasing the	Normothermia.

	temperature on the management system by 1 °C per day until the patient reached 36 °C.	
Moler et al. 2015	Children who were assigned to therapeutic hypothermia were pharmacologically paralyzed and sedated, and a Blanketrol III temperature management unit (Cincinnati Sub-Zero) was used, with blankets applied anteriorly and posteriorly, to achieve and maintain a core temperature of 33.0°C (range, 32.0 to 34.0) for 48 hours. The children were then rewarmed over a period of 16 hours or longer to a target temperature of 36.8°C (range, 36.0 to 37.5); this temperature was actively maintained throughout the remainder of the 120-hour intervention period.	Identical care except that the core temperature was actively maintained with the cooling unit at 36.8°C (range, 36.0 to 37.5) for 120 hours.
Moler et al. 2017	Targeted temperature management was active- ly maintained for 120 hours in each group. Pa- tients who were assigned to therapeutic hypo- thermia were pharmacologically paralyzed and sedated, and a Blanketrol III temperature-man- agement unit (Cincinnati Sub-Zero) was used, with blankets applied anteriorly and posteriorly, to achieve and maintain a core temperature of 33.0°C (range, 32.0 to 34.0) for 48 hours. The patients were then rewarmed over a period of 16 hours or longer to a target temperature of 36.8°C (range, 36.0 to 37.5); this temperature was actively maintained throughout the remainder of the 120- hour intervention period.	Identical care except that the core temperature was actively maintained with the temperature- management unit at 36.8°C (range, 36.0 to 37.5) for 120 hours.
Scholefield et al. 2015	TTM (32–34◦C) was initiated in the PICU with the use of servo-controlled water blanket cooling mattresses (Blanketroll II, Cincinnati Sub Zero, OH, USA) to reduce temperature between 32 and 34 ◦ C for 24 h followed by controlled rewarming, by 0.5 ◦ C every 2 h, and 37 ◦ C.	Non-TTM practice followed recommendations to avoid hyperthermia (>38°C).
van Zellem et al. 2015	Hypothermia was achieved by administering a bolus of cold fluids and applying external cooling using a mattress with Blanketrol® III (Cincinnati Sub-Zero Products, Inc., Sharonville, OH, USA). The target temperature is 32–34 ◦ C for 24 h following ROSC, after which they were rewarmed passively at a rate of 0.5 ◦ C per 2 h. The target temperature must have been reached for MTH to be effective.	Children in whom the target temperature range was not reached

Legend: ECMO, ExtraCorporeal Membrane Oxygenation; NS, not specified; ROSC, return of spontaneous circulation; TTM, Targeted temperature management.

Table S3. Adverse events.

Adverse Event Type	No. of Studies	No. of Cases in TTM Group	No. of Cases in Non-TTM Group	OR (95%CI)	*p* Value	I^2 Statistic
**Adverse events for 7 days after cardiac arrest**						
Serious arrythmias	2	42/314	37/297	1.10 (0.68, 1.77)	0.70	0%
Asystole	2	9/314	10/297	0.84 (0.33, 2.09)	0.70	0%
Atrial (SVT, AF, JET)	2	11/314	6/297	1.79 (0.65, 4.90)	0.26	0%
PEA	2	4/314	5/297	0.79 (0.22, 2.80)	0.72	0%
Ventricular (VF, VT, TPD)	2	13/314	12/297	1.03 (0.46, 2.30)	0.93	0%
Re-arrest not specified	1	5/40	36/141	0.42 (0.15, 1.14)	0.09	NA
Other	2	18/314	11/297	1.62 (0.75, 3.50)	0.22	7%
**Adverse events for 14 days after cardiac arrest**						
Cardiac tachyarrhythmia (all)	1	5/29	9/50	0.95 (0.28, 3.16)	0.93	NA
Ventricular	1	4/29	7/50	0.98 (0.26, 3.69)	0.98	NA
Supraventricular	1	1/29	2/50	0.86 (0.07, 9.89)	0.90	NA
Subsequent cardiac arrest	1	2/29	13/50	0.21 (0.04, 1.01)	0.05	NA
Pulmonary edema	1	9/29	17/50	0.87 (0.33, 2.33)	0.79	NA
Renal replacement	1	14/29	16/50	1.98 (0.77, 5.08)	0.15	NA
Hepatic dysfunction	1	2/29	8/50	0.39 (0.08, 1.97)	0.25	NA
Venous thromboembolism	1	0/29	1/50	0.56 (0.02, 14.18)	0.72	NA
Arterial occlusion	1	1/29	1/50	1.75 (0.11, 29.08)	0.70	NA
Cerebral herniation	1	1/29	2/50	0.86 (0.07, 9.89)	0.90	NA
Clinically significant hemorrhage						
Intracranial	1	1/29	3/50	0.56 (0.06, 5.64)	0.62	NA
Gastrointestinal tract	1	2/29	1/50	3.63 (0.31, 41.89)	0.30	NA
Open sternotomy	1	3/29	5/50	1.04 (0.23, 4.70)	0.96	NA
Vascular access	1	5/29	2/50	5.00 (0.90, 27.69)	0.07	NA
Pulmonary	1	0/29	4/50	0.18 (0.01, 3.37)	0.25	NA
Clinically significant infection						
Pneumonia	1	6/29	11/50	0.92 (0.30, 284)	0.89	NA
Septicemia	1	7/29	6/50	2.33 (0.70, 7.78)	0.17	NA
Urinary tract infection	1	2/29	1/50	3.63 (0.31, 41.89)	0.30	NA
Peritonitis	1	0/29	1/50	0.56 (0.02, 14.18)	0.72	NA
Wound	1	2/29	4/50	0.85 (0.15, 4.96)	0.86	NA
**Adverse events for 30 days after cardiac arrest**						
Bradycardia	1	13/25	10/39	3.14 (1.08, 9.10)	0.03	NA
Coagulopathy	1	11/25	8/39	3.04 (1.01, 9.22)	0.05	NA
Electrolyte imbalance	1	13/25	21/39	0.93 (0.34, 2.54)	0.89	NA
Hypokalemia	1	12/25	16/39	1.33 (0.48, 3.65)	0.58	NA
Hypocalcemia	1	5/25	11/39	0.64 (0.19, 2.12)	0.46	NA
Hypophosphatemia	1	4/25	4/39	1.67 (0.38, 7.38)	0.50	NA
Hypomagnesemia	1	1/25	5/39	0.28 (0.03, 2.58)	0.26	NA
Infection	1	0.25	3/39	0.20 (0.85, 1.98)	0.30	NA

Legend: CI = Confidence interval; NA = Not applicable; OR = Odds ratio; TTM = Targeted temperature management.

Table S4. Cardiac etiology of cardiac arrest in TTM and not TTM group.

Adverse Event Type	No. of Studies	No. of Cases in TTM Group	No. of Cases in Non-TTM Group	OR (95%CI)	*p* Value	I^2 Statistic
OHCA	4	79/337 (23.4%)	360/894 (40.3%)	0.97 (0.70, 1.35)	0.85	6%
IHCA	0	-	-	-	-	-
Total	7	106/421 25.2%)	405/1,113 (36.4%)	1.05 (0.78, 1.42)	0.76	0%

Legend: CI = Confidence interval; NA = Not applicable; OR = Odds ratio; TTM = Targeted temperature management.

Table S5. The Grading of Recommendations Assessment, Development and Evolution (GRADE) approach.

Certainty assessment							Summary of findings				
							Study event rates (%)			Anticipated absolute effects	
**Participants (studies) Follow up**	**Risk of bias**	**Inconsistency**	**Indirectness**	**Imprecision**	**Publication bias**	**Overall certainty of evidence**	**With non-TTM**	**With TTM**	**Relative effect (95% CI)**	**Risk with non-TTM**	**Risk difference with TTM**
**Survival to hospital discharge (randomized trials)**											
619 (2 RCTs)	not serious	serious	not serious	not serious	none	⊕⊕⊕◯ MODERATE	177/300 (59.0%)	170/319 (53.3%)	**OR 0.80** (0.58 to 1.10)	590 per 1000	**55 fewer per 1000** (from 135 fewer to 23 more)
**Survival to hospital discharge (non-randomized studies)**											
1387 (8 observational studies)	serious	not serious	not serious	not serious	none	⊕◯◯◯ VERY LOW	428/1042 (41.1%)	150/345 (43.5%)	**OR 1.17** (0.90 to 1.51)	411 per 1000	**38 more per 1000** (from 25 fewer to 102 more)
**Survival (randomized trials) (follow up: mean 6 months)**											
624 (2 RCTs)	not serious	serious	not serious	not serious	none	⊕⊕⊕◯ MODERATE	122/303 (40.3%)	144/321 (44.9%)	**OR 1.23** (0.89 to 1.70)	403 per 1000	**51 more per 1000** (from 28 fewer to 131 more)
**Survival (randomized trials) (follow up: mean 1 years)**											
614 (2 RCTs)	not serious	serious	not serious	not serious	none	⊕⊕⊕◯ MODERATE	113/297 (38.0%)	138/317 (43.5%)	**OR 1.28** (0.92 to 1.77)	380 per 1000	**60 more per 1000** (from 19 fewer to 140 more)
**Survival with WABS-II score ≥ 70 points (randomized trials) (follow up: mean 1 years)**											

**Certainty assessment**							**Summary of findings**				
517 (2 RCTs)	not serious	serious	not serious	not serious	none	⊕⊕⊕◯ MODERATE	63/246 (25.6%)	75/271 (27.7%)	**OR 1.17** (0.90 to 1.51)	256 per 1000	**31 more per 1000** (from 20 fewer to 86 more)

CI: Confidence interval; OR: Odds ratio; RCT: Randomized trial; VABS: Vineland adaptive behavior scale.

Study or Subgroup	TTM Mean	TTM SD	TTM Total	Non-TTM Mean	Non-TTM SD	Non-TTM Total	Weight	Mean Difference IV, Random, 95% CI
Chang 2016	14.5	1.3	81	7.5	2.3	582	11.7%	7.00 [6.66, 7.34]
Cheng 2018	0.8	0.6	26	0.4	0.3	49	11.7%	0.40 [0.15, 0.65]
Fink 2010	6	6.6	40	6	6.2	141	8.8%	0.00 [−2.29, 2.29]
Meert 2016	0.3	0.2	26	0.2	0.2	28	11.7%	0.10 [−0.01, 0.21]
Moler 2015	3.7	1.6	155	2.7	1.1	140	11.7%	1.00 [0.69, 1.31]
Moler 2016	3.1	1.3	46	4.3	1.8	28	11.3%	−1.20 [−1.97, −0.43]
Moler 2017	2.2	0.9	166	1.9	1	163	11.7%	0.30 [0.09, 0.51]
Scholefield 2015	2.2	1.7	38	1.5	1.2	35	11.4%	0.70 [0.03, 1.37]
van Zellem 2015	6.5	5.1	63	6.4	6.3	137	10.0%	0.10 [−1.54, 1.74]
**Total (95% CI)**			**641**			**1303**	**100.0%**	**0.98 [−0.38, 2.34]**

Heterogeneity: Tau^2 = 4.11; Chi^2 = 1490.61, df = 8 (P < 0.00001); I^2 = 99%
Test for overall effect: Z = 1.41 (P = 0.16)

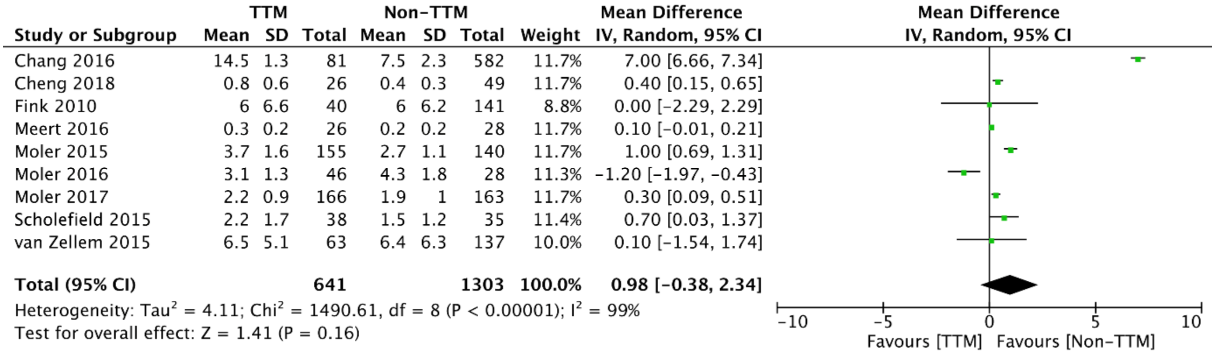

Figure S1. Forest plot of patients age in TTM and not TTM group. The center of each square represents the weighted mean differences for individual trials, and the corresponding horizontal line stands for a 95% confidence interval. The diamonds represent pooled results.

Study or Subgroup	TTM Events	TTM Total	Non-TTM Events	Non-TTM Total	Weight	Odds Ratio M-H, Fixed, 95% CI
Chang 2016	25	81	199	582	18.3%	0.86 [0.52, 1.42]
Cheng 2018	12	26	33	49	6.7%	0.42 [0.16, 1.10]
Doherty 2009	16	29	23	50	4.1%	1.44 [0.58, 3.62]
Fink 2010	24	40	80	141	7.7%	1.14 [0.56, 2.34]
Lin 2013	10	15	18	28	2.3%	1.11 [0.30, 4.17]
Lin 2018	21	25	28	39	1.9%	2.06 [0.58, 7.39]
Meert 2016	18	26	14	28	2.3%	2.25 [0.74, 6.86]
Moler 2015	102	155	94	140	18.4%	0.94 [0.58, 1.53]
Moler 2016	28	46	22	28	5.8%	0.42 [0.14, 1.25]
Moler 2017	97	166	99	163	22.6%	0.91 [0.58, 1.41]
Scholefield 2015	17	38	8	35	2.5%	2.73 [0.99, 7.54]
van Zellem 2015	43	63	67	137	7.3%	2.25 [1.20, 4.21]
**Total (95% CI)**		**710**		**1420**	**100.0%**	**1.08 [0.89, 1.33]**
Total events	413		685			

Heterogeneity: Chi^2 = 19.76, df = 11 (P = 0.05); I^2 = 44%
Test for overall effect: Z = 0.80 (P = 0.43)

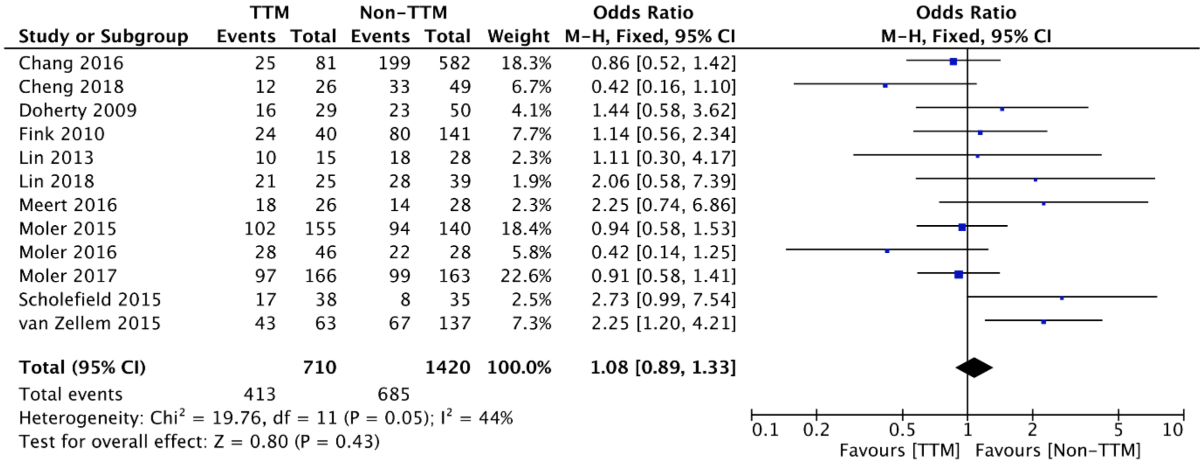

Figure S2. Forest plot of patients gender (male) in TTM and not TTM group. The center of each square represents the weighted odds ratios for individual trials, and the corresponding horizontal line stands for a 95% confidence interval. The diamonds represent pooled results.

Study or Subgroup	TTM Events	TTM Total	Non-TTM Events	Non-TTM Total	Weight	Odds Ratio M-H, Fixed, 95% CI
**1.5.2 Cardiac**						
Chang 2016	44	81	298	582	40.2%	1.13 [0.71, 1.81]
Doherty 2009	21	29	34	50	8.3%	1.24 [0.45, 3.39]
Fink 2010	5	40	11	141	5.1%	1.69 [0.55, 5.18]
Lin 2013	1	15	0	28	0.4%	5.90 [0.23, 153.98]
Moler 2015	14	155	18	140	20.8%	0.67 [0.32, 1.41]
Scholefield 2015	4	38	1	35	1.1%	4.00 [0.42, 37.66]
van Zellem 2015	17	63	43	137	23.9%	0.81 [0.42, 1.57]
**Subtotal (95% CI)**		**421**		**1113**	**100.0%**	**1.05 [0.78, 1.42]**
Total events	106		405			
Heterogeneity: Chi^2 = 5.32, df = 6 (P = 0.50); I^2 = 0%						
Test for overall effect: Z = 0.30 (P = 0.76)						
**Total (95% CI)**		**421**		**1113**	**100.0%**	**1.05 [0.78, 1.42]**
Total events	106		405			

Heterogeneity: Chi^2 = 5.32, df = 6 (P = 0.50); I^2 = 0%
Test for overall effect: Z = 0.30 (P = 0.76)
Test for subgroup differences: Not applicable

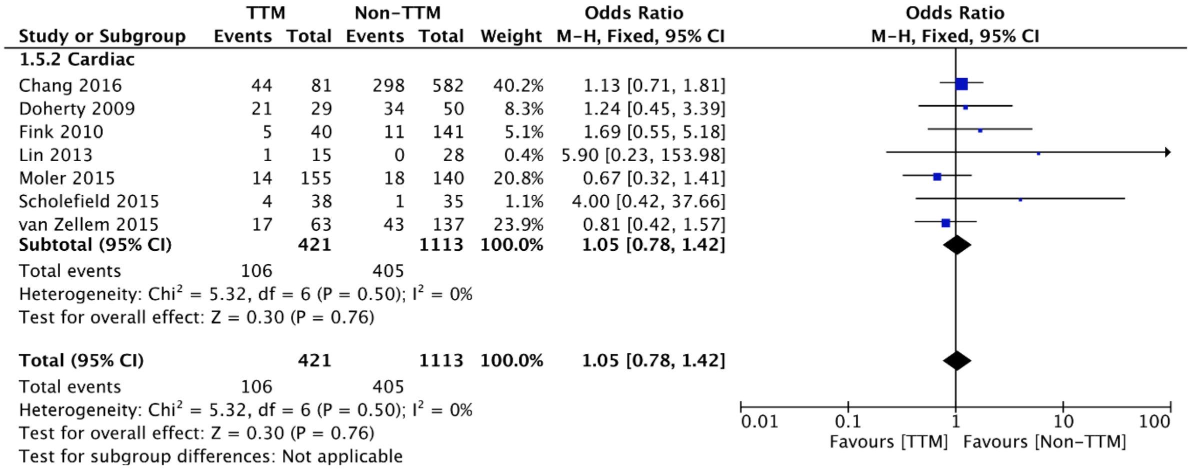

Figure S3. Forest plot of cardiac etiology of cardiac arrest in TTM and not TTM group. The center of each square represents the weighted odds ratios for individual trials, and the corresponding horizontal line stands for a 95% confidence interval. The diamonds represent pooled results.

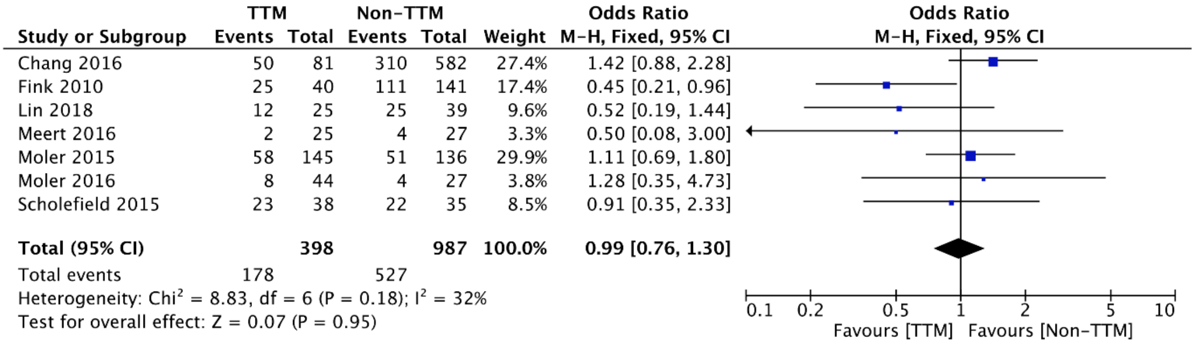

Figure S4. Forest plot of witnessed cardiac arrest in TTM and not TTM group. The center of each square represents the weighted odds ratios for individual trials, and the corresponding horizontal line stands for a 95% confidence interval. The diamonds represent pooled results.

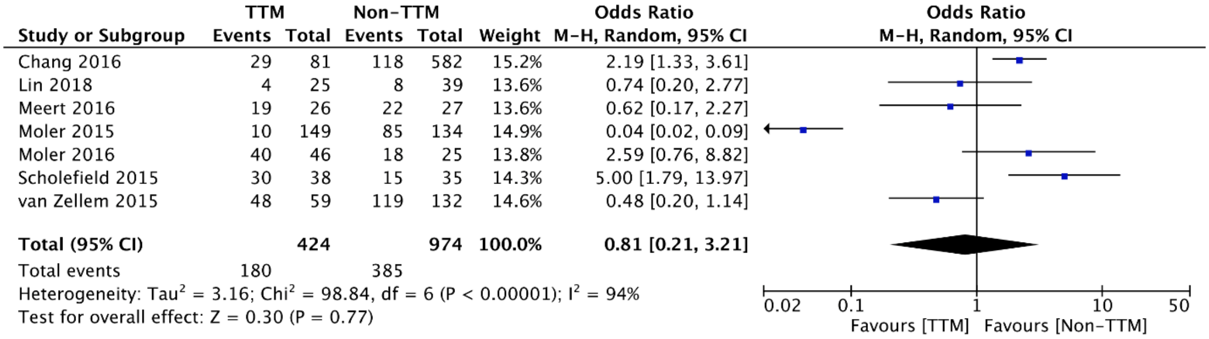

Figure S5. Forest plot of bystander cardiopulmonary resuscitation in TTM and not TTM group. The center of each square represents the weighted odds ratios for individual trials, and the corresponding horizontal line stands for a 95% confidence interval. The diamonds represent pooled results.

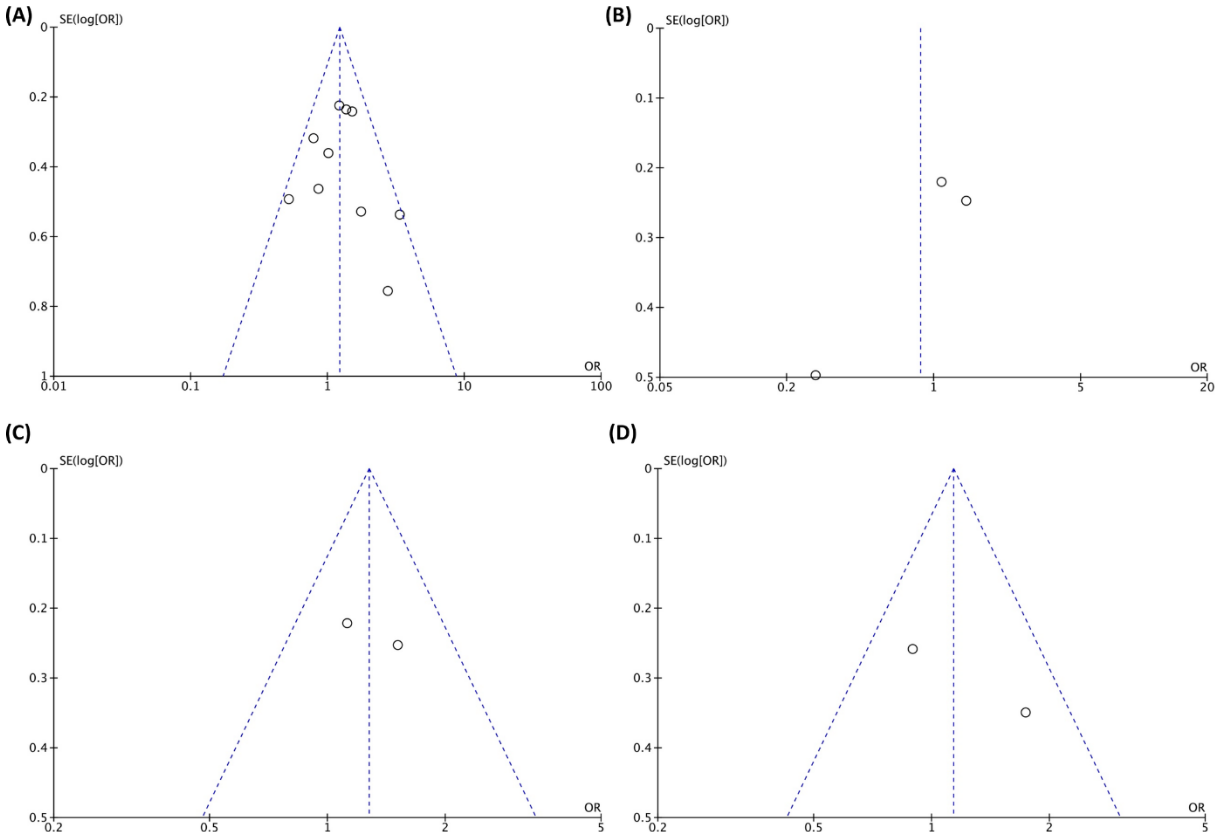

Figure S6. Funnel plot of odds ratio (OR) with standard error: (A) survival to hospital discharge, (B) survival rate in 6-months follow-up; (C) survival rate in 1-year follow-up; (D) survival with VABS-II score ≥ 70 points at 1-year follow-up. Data from each modality are plotted against their standard error (SE). Solid line = summary estimate of the odds ratio; dashed line = 95%CI confidence limits around the OR.

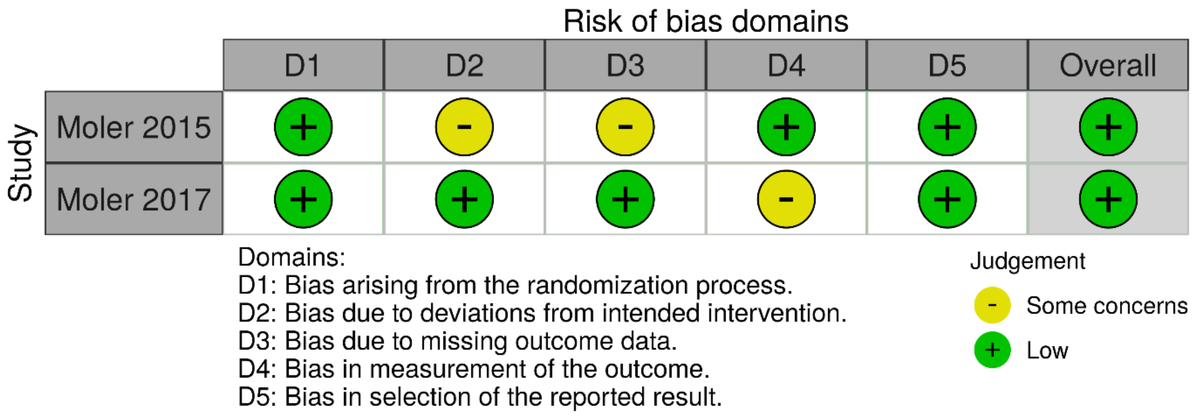

Figure S7. A summary table of review authors' judgements for each risk of bias item for each randomized study.

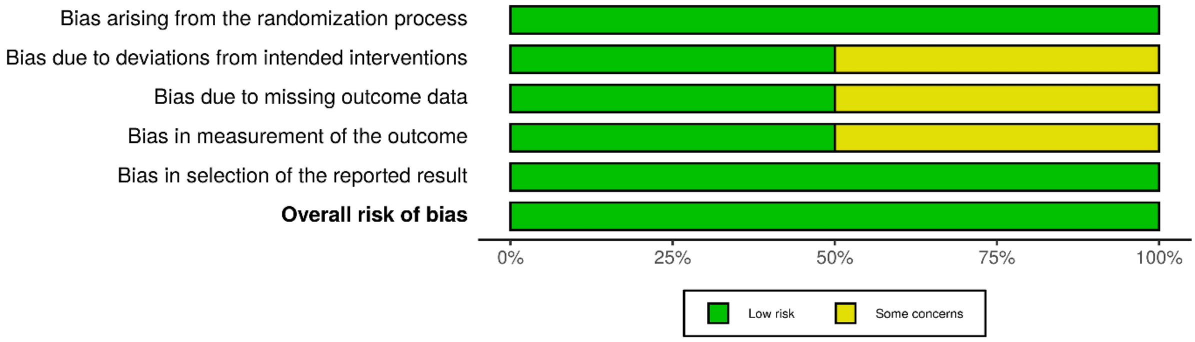

Figure S8. A plot of the distribution of review authors' judgements across randomized studies for each risk of bias item.

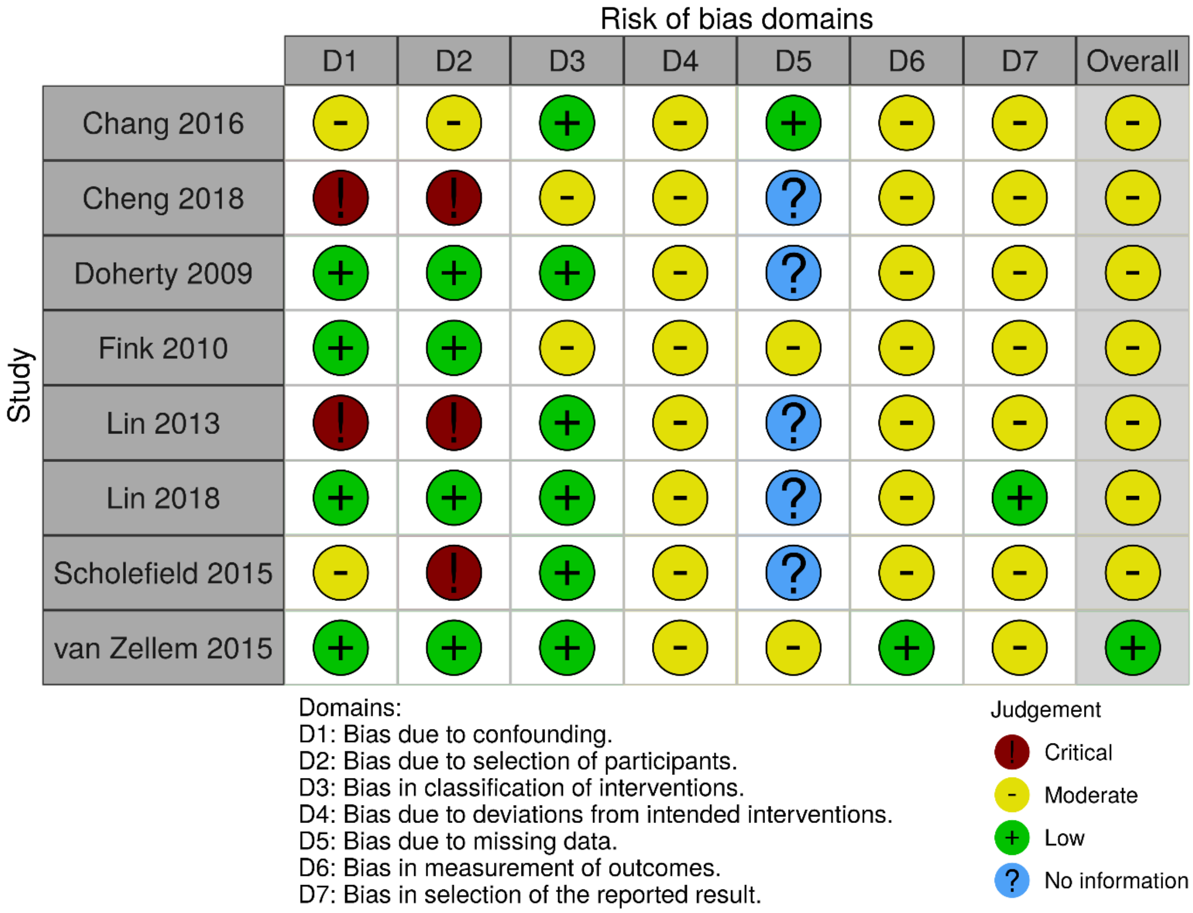

Figure S9. A summary table of review authors' judgements for each risk of bias item for each non-randomized study.

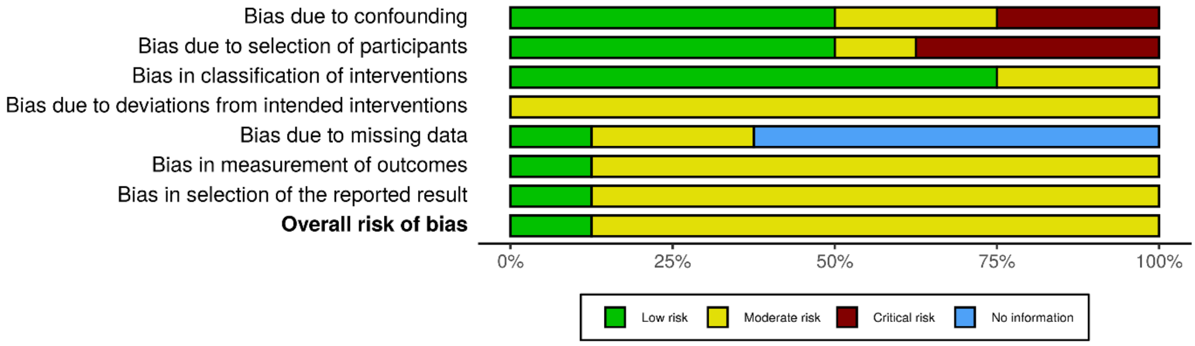

Figure S10. A summary table of review authors' judgements for each risk of bias item for each non-randomized study.
